# It Takes Two: Endothelial-Perivascular Cell Cross-Talk in Vascular Development and Disease

**DOI:** 10.3389/fcvm.2018.00154

**Published:** 2018-10-30

**Authors:** Mark Sweeney, Gabor Foldes

**Affiliations:** ^1^Cardiovascular Division, National Heart and Lung Institute, Imperial College London, London, United Kingdom; ^2^Heart and Vascular Center, Semmelweis University, Budapest, Hungary

**Keywords:** cell-cell interaction, vascular development, endothelial, pericytes, perivascular, vascular dysfunction

## Abstract

The formation of new blood vessels is a crucial step in the development of any new tissue both during embryogenesis and *in vitro* models as without sufficient perfusion the tissue will be unable to grow beyond the size where nutrition and oxygenation can be managed by diffusion alone. Endothelial cells are the primary building block of blood vessels and are capable of forming tube like structures independently however they are unable to independently form functional vasculature which is capable of conducting blood flow. This requires support from other structures including supporting perivascular cells and the extracellular matrix. The crosstalk between endothelial cells and perivascular cells is vital in regulating vasculogenesis and angiogenesis and the consequences when this is disrupted can be seen in a variety of congenital and acquired disease states. This review details the mechanisms of vasculogenesis *in vivo* during embryogenesis and compares this to currently employed *in vitro* techniques. It also highlights clinical consequences of defects in the endothelial cell—pericyte cross-talk and highlights therapies which are being developed to target this pathway. Improving the understanding of the intricacies of endothelial—pericyte signaling will inform pathophysiology of multiple vascular diseases and allow the development of effective *in vitro* models to guide drug development and assist with approaches in tissue engineering to develop functional vasculature for regenerative medicine applications.

## Introduction

The processes of vasculogenesis and angiogenesis play vital roles in embryonic development and vascular homeostasis during adulthood. The endothelial cell is the most basic building block of blood vessels and the growth factor mediated proliferation and migration of these cells is responsible for forming the complex vascular networks within the body. Endothelial cells do not perform these tasks in isolation and their interaction with supporting perivascular cells is one of the key processes during the formation of new blood vessels to ensure that durable vessels are formed which can support blood flow. This review will discuss the embryological origins and interactions of endothelial cells and perivascular cells and compare this with current *in vitro* approaches used to model this interaction. In addition, it will discuss how interruption of this interaction causes a variety of genetic and acquired diseases and how novel approaches to co-culture may help to develop our understanding of this area and provide potential therapeutic options in the future.

## Multicellular interactions in embryogenesis

### Building blocks for new vessels

The process of creating vascular networks involves two sequential steps: vasculogenesis, the *de novo* formation of blood vessels from progenitor cells, and angiogenesis the migration, branching, and pruning of existing blood vessels to form complex vascular networks and capillary beds (1). The endothelial cell is the most basic building block of new blood vessels and the processes of angiogenesis and vasculogenesis both require the proliferation and migration of these cells to under perfused tissues. This must be followed by the formation of strong connections between adjacent cells and the extra-cellular matrix (ECM) to create a durable conduit which can support blood flow. In the developing embryo there are multiple interactions between the cell and its environment responsible for controlling this process (2). This includes interactions between neighboring endothelial cells, between endothelial cells and surrounding support cells as well as the paracrine effects of growth factors released into the ECM. In addition, these newly developing vessels respond to changes in the extracellular environment including the composition of the ECM and relative levels of hypoxia or nutritional deficiencies of surrounding cells (3).

#### Endothelial cells

During embryogenesis the first recognizable blood vessels occur in the yolk sac as groups of cells expressing endothelial markers including vascular endothelial growth factor receptor (VEGFR), VE-cadherin and CD31 (1, 4). These primitive endothelial cells are derived from the mesodermal layer of the embryo. They migrate to form aggregates of cells known as blood islands which are capable differentiating toward either haematopoietic or angioblastic lineages (5). As these cells begin to differentiate they align with angioblastic cells on the outside of the blood islands and haematopoietic cells in the central core. Angioblasts in the outer lining flatten and form intercellular connections to create a circumferential layer of primitive endothelial cells which is the first stage in vessel formation (1).

The formation of these blood islands in the mesoderm is controlled by growth factors released from the endodermal layer. Hedgehog signaling via the bone morphogenic protein-4 (BMP-4) pathway is one of the earliest steps that initiates endothelial differentiation from multipotent mesodermal cells and is vital in early vascular development (6–8). Fibroblast growth factors (FGF) stimulation of these cells induces the expression of early endothelial markers. The FGF driven expression of VEGFR (9–11) is an essential step in sensitizing the cells to the potent angiogenic growth factor vascular endothelial growth factor (VEGF) which is one of the key growth factors in promoting angiogenesis (12, 13).

As the blood vessel matures the endothelial layer forms a confluent monocellular layer in contact with the blood. This functions as barrier to prevent the widespread extravasation of blood and fluid however also needs to be sufficiently permeable to enable the passage of required gases, nutrient and leukocytes into the perivascular space when required. VE-cadherin one of the earliest markers expressed on the surface of developing endothelial cells. It forms part of the adherens junctions between endothelial cells to begin the formation of the monolayer. Further control of the permeability is mediated by the formation of tight junctions which are formed from claudins, occludins and junctional adhesion molecules which are upregulated as the endothelial cell matures (14).

#### Perivascular cells

Perivascular or mural cells were first described histologically as cells closely associated to the endothelial layer of blood vessel and are found in all organs throughout the body. They are a phenotypically diverse family of cells with a variety of roles depending upon the anatomical location and function of the vessel (15). They can be divided into two main categories: vascular smooth muscle cells and pericytes although much heterogeneity exists within these groups.

Vascular smooth muscle cells are associated with larger conduit vessels such as arteries and veins and are separated from the endothelial layer by the basement membrane and the inner elastic lamina. They have greater expression of contractile proteins such as α-smooth muscle actin (α-SMA), adopt a stellate shape and line up in a circumferential pattern around the vessel. Pericytes, in contrast, are associated with small caliber capillaries where they are embedded in the same basement membrane as the endothelial layer. They have an elongated and flattened shape and are orientated along the long axis of the vessel with multiple finger-like projection extending through the basement membrane to make direct contact with the endothelial layer (15). These connections have a peg and socket arrangement that enables signals to be passed directly between the pericyte and endothelial layers. A single pericyte can interact with multiple endothelial cell and the relative density of pericytes surrounding a blood vessel depends upon anatomical location and function of the vessel. In skeletal muscle there is approximately a 1:100 relationship between pericytes and endothelial cells (16) whereas in the brain and the retina there is a 1:1 relationship where it is thought to play a role in maintaining the blood brain barrier (17–19). Their position in contact with multiple endothelial cells can be advantageous in co-ordinating the response of a population of endothelial cells along the vessel as intercellular signaling can be propagated to multiple cells in the endothelial layer through a single pericyte (15, 17).

Debate exists regarding the characteristics which define a perivascular cell and thus a precise definition remains elusive. Consequently, there is no single specific marker to define this population. Instead, a combination of cell surface markers are typically used and these may have variable expression depending upon the anatomical location and angiogenic state of the cell (20, 21). Common markers which are expressed in perivascular cells include neuron-glial antigen-2 (NG2), CD146, α-smooth muscle actin (α-SMA), and platelet derived growth factor receptor-β (PDGFR-β). Endothelial and haematopoietic specific markers such as CD31, VE-cadherin, von Willebrand factor and CD45 should be absent (15, 22–25).

**Figure 1:**
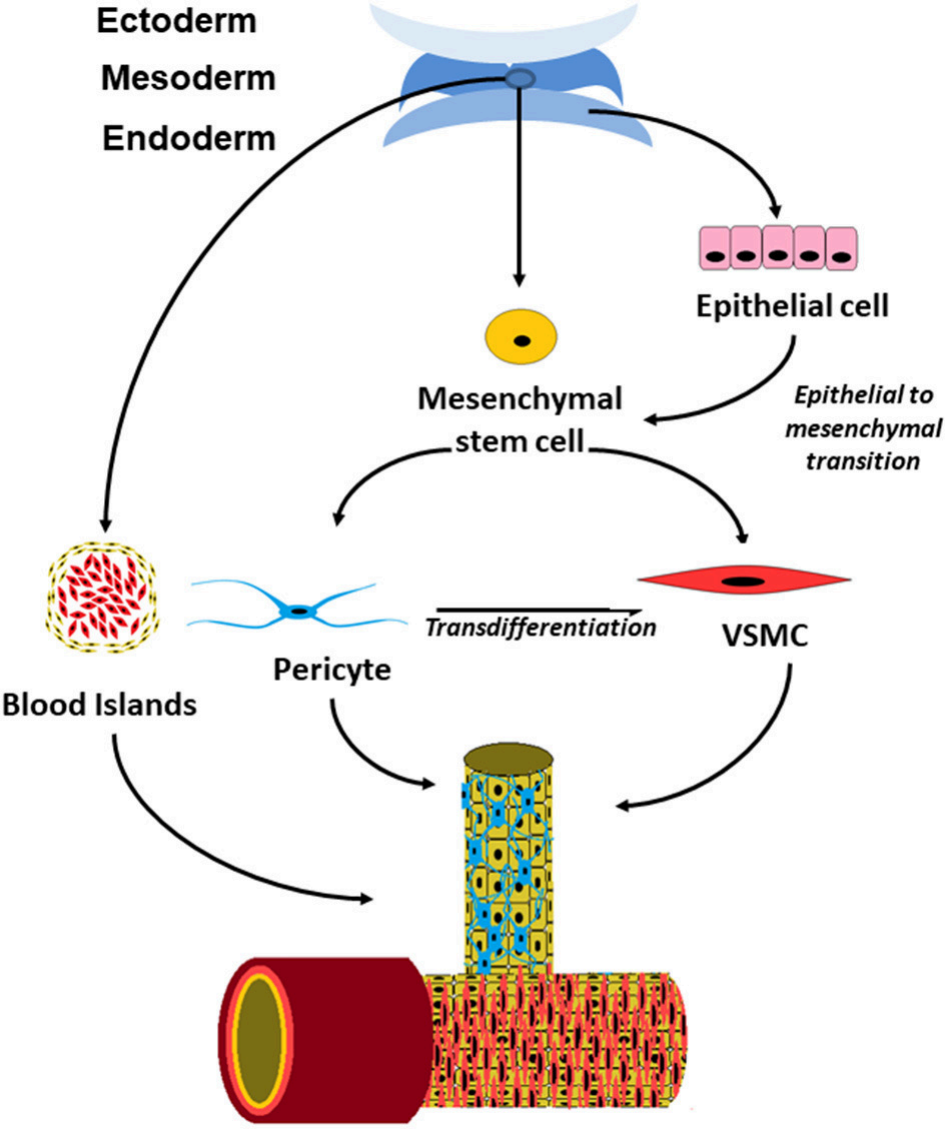
Schematic drawing showing the origin of perivascular cells in embryonic development and postnatal vessels.

The precise origin of perivascular cells is more controversial than that of endothelial cells and there appears to be more than a single origin for these cells (Figure 1). Perivascular cells in the bowel, lung and liver have been found to have an endodermal lineage which initially develops toward an epithelial fate and subsequently undergo epithelial to mesenchymal transition into pericytes (16). In the brain, they are derived from neural crest cells (26) and vessel such as the aorta have pericytes of multiple different origins within a single vessel (16).

Perivascular cells were once thought to only provide mechanical support to the vessel however it is now clear that it has a variety of functions within the vasculature. The contractile properties of perivascular cells enable regulation of blood supply by altering the vessel diameter in response to vasoactive substances (21). In the central nervous system, where they are significantly more abundant, they are thought to play a role in the control of cerebral perfusion and restricting the permeability of the blood brain barrier (15, 21, 27). They may also play a role in tissue repair as they have the ability to transdifferentiate into fibroblast in response to injury to the surrounding tissue (16, 28) Finally, they also have an important role in intercellular signaling both to endothelial cells and to the tissues surrounding the blood vessels.

### Crosstalk between perivascular and endothelial cells is key during early vasculogenesis

**Figure 2:**
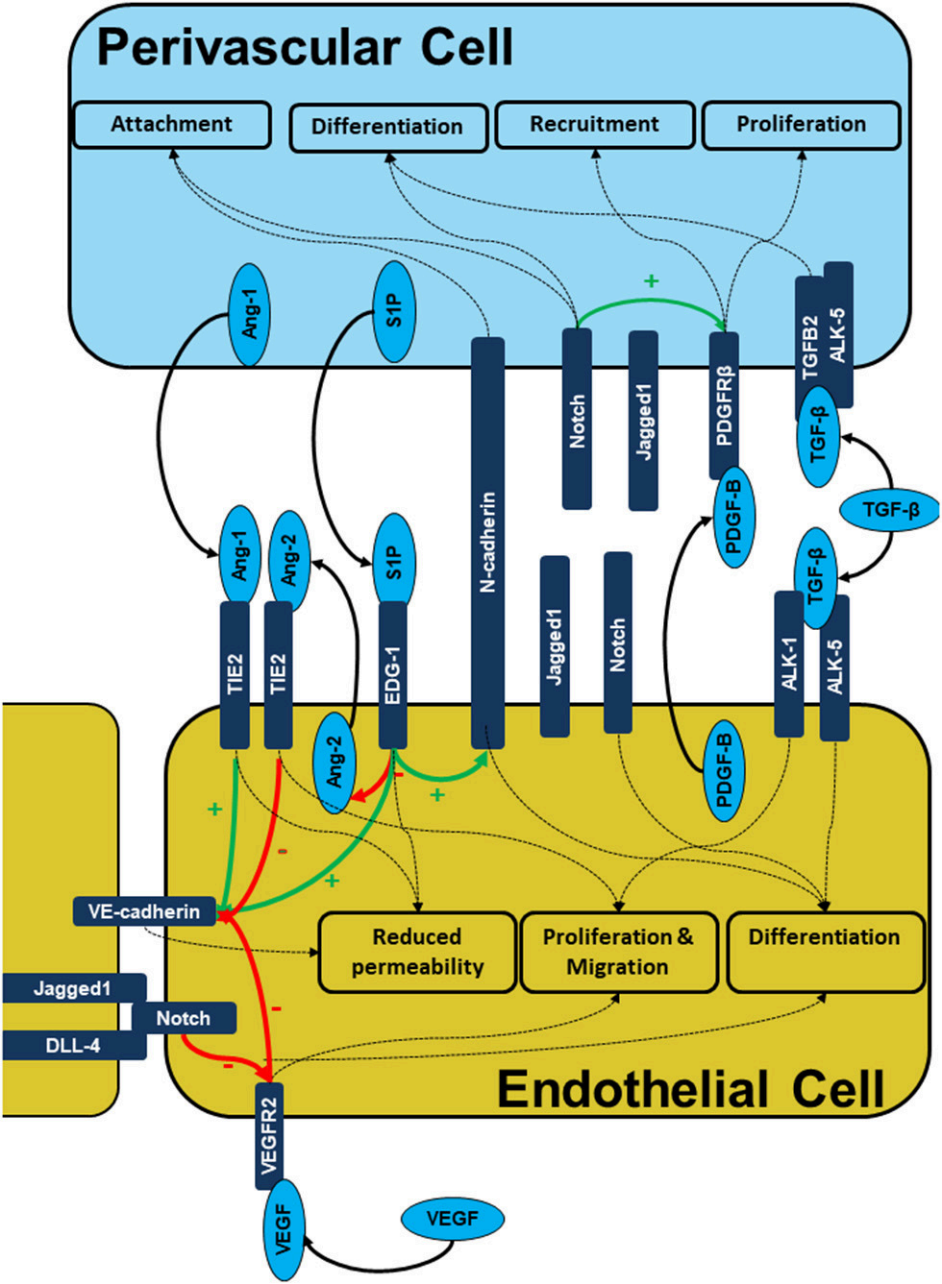
Signaling during endothelial-pericyte cross-talk. The figure illustrates the intercellular signaling responsible for cell recruitment, differentiation, and maturation as well as vessel stability is built on multiple receptor complexes. PDGF-B/PDGFRB2, S1P/EDG-1, ANG1/2/TIE2, Cadherin, and Notch mediated signals are prerequisites of endothelial -pericyte cross-talk, cell recruitment and subsequent vessel stabilization.

Signaling between the endothelial and perivascular cell layers is mediated by the release of growth factors, direct cell-cell contact at peg and socket connections and modulation of the ECM (Figure 2). Perivascular cell are recruited to the developing vessel as the vessel matures. Perivascular cells coating the vessel have an anti-angiogenic effect which stabilizes the vessel and limits further proliferation and migration of the endothelial cells. This is mediated by a number of signaling molecules released by the perivascular cells which act on the endothelial layer including Angiopoietins, sphingosine-1-phosphate as well as contact inhibition largely mediated by the Notch family.

#### VEGF

The expression of VEGF receptor on primitive endothelial cells is one of the most important steps in vasculogenesis as this enables cells to respond to the potent mitogenic and chemotactic effects of VEGF (10, 29). The importance VEGF signaling pathway in early vasculogenesis is highlighted by VEGF knockout mice which die early in development without any organized vasculature present (30, 31). VEGF-A is the prototypical member of the VEGF family and is coded for by the VEGFA gene on chromosome 6. It is released by multiple cells throughout the body typically in response to hypoxia or hypoperfusion of a tissue (32).

VEGF receptors are transmembrane tyrosine kinase receptors denoted VEGFR1-R3. The most important signaling receptor in vasculogenesis and angiogenesis is the VEGFR2 which is a relatively low affinity receptor with high intracellular tyrosine kinase activity when activated. Knockout of this receptor has the same effects as VEGF-A knockouts with early embryonic lethality due to lack of organized vasculature. In contrast, VEGFR1 has a much higher affinity for VEGF-A however minimal intracellular tyrosine kinase activity upon activation (32). Knockout of the VEGFR1 receptor causes unchecked endothelial hyperplasia (33) however selective knockout of the tyrosine kinase domain of the VEGFR1 has minimal effect on vascular development. This demonstrates that this receptor acts largely as ligand trap to sequester VEGF and reduce VEGFR2 signaling and therefore angiogenesis (33).

Intracellularly activation of VEGFR2 is propagated by a number of downstream signaling pathways. This is classically via ERK signaling, however, a variety of non-canonical pathways are also known to play a role (34). Stimulation of this pathway induces endothelial proliferation and differentiation including the expression of cell adhesion molecules such as VE-cadherin which form junctional complexes between the endothelial cells and creates the tubular structure from which further blood vessel development can occur (35, 36). Control of VEGF receptor expression is maintained by exogenous signaling including concentration of FGF and transforming growth factor-β (TGF-β) as well as by negative feedback loops within the cell to internalize and degrade receptors to prevent overstimulation of the VEGFR2 pathway and excessive neovascularisation with immature and permeable vessels as is seen in VEGFR1 knockout mice.

As perivascular cells differentiate they begin to express VEGF which is expressed in response to hypoxia and TGF-β signaling (28, 37). The isoform produced by perivascular cells is most commonly VEGF-A^165^ which binds to heparin sulfate proteoglycans on the cell surface, therefore, remaining associated with the cells (37). This local release of VEGF is important in promoting endothelial cell survival and stabilizing newly formed vessels, however, does not set up a concentration gradient to encourage endothelial cell migration as occurs when more soluble isoforms are released (37, 38). Pericytes also have VEGFR1 on the cell surface which binds VEGF and sequesters it from the VEGFR2 on the endothelial cells, therefore, prevents the initiation of angiogenesis in mature, quiescent vessels (39).

#### TGF-β

The transforming growth factor-β family of signaling molecules consists of more than thirty molecules which have a diverse range of targets. TGF-β is the most well characterized member and is expressed by multiple cells throughout the body including both endothelial and perivascular cells. When secreted by these cells it is associated with specific TGF-β binding proteins which maintain it in an inactivated form bound to the extracellular matrix (ECM) (40). Various mechanisms can release and activate TGF-β1 including the action of proteases such as matrix metalloproteinases (MMPs) which are produced by endothelial and perivascular cells when they come into contact. Once activated TGF-β has a variety of receptors on the surface of endothelial cells and pericytes (23, 41, 42).

The effect of TGF-β is highly context dependent and varies with the local concentration of TGF-β as well as the relative expression of receptors on the cell surface of the target cells (43, 44). The two most important TGF- β receptors in angiogenesis are Activin like kinase-1 (ALK-5) which is expressed on both endothelial cells and perivascular cells and Activin like kinase-1 (ALK-1) which is restricted mainly to endothelial cells. These two receptors have opposing effects on the response of the cell to TGF-β. ALK-5 stimulation causes down regulation of proangiogenic VEGFR-2 and upregulation of the antiangiogenic VEGFR-1 leading to reduced proliferation and increased differentiation of endothelial cells (45, 46). Similarly, ALK-5 activation causes differentiation of the perivascular cells and promotes release of ECM proteins such as fibronectin from both cell types (40). Overall this encourages formation of quiescent mature vessels with significant perivascular cell coating and a well-developed stable ECM. In contrast, ALK-1 signaling which is preferentially activated at lower concentrations of TGF-β increases proliferation of endothelial cells and encourages angiogenesis to proceed (45, 47, 48).

#### Platelet derived growth factor

Platelet derived growth factor (PDGF) is released mainly by endothelial cells and binds to the PDGFR-β on perivascular cells. Activation of this receptor stimulates proliferation of perivascular cells and acts as a potent chemoattractant causing cells to migrate toward expanding endothelial cell populations (49, 50). Absence of PDGF or PDGFR- β in mice is lethal in late gestation due to large, permeable, dilated vessels with microaneurysm formation, microvascular leakage and oedema throughout the embryo (49). Histologically these mice have widespread deficiency of perivascular cells coating the blood vessels and there is endothelial cell hyperplasia at the site of microaneurysms formation. This demonstrates the role that recruitment of perivascular cells to the perivascular niche plays in stabilizing developing vessels and reducing proliferation and angiogenesis in fully formed mature vessels (24).

VEGF signaling on perivascular cells via the VEGFR2 supresses the response of the cell to stimulation by PDGF-B by phosphorylation of the PDGFR-β receptor. This reduces the maturation and migration of perivascular cells to the site blood vessels undergoing of active angiogenesis and enables endothelial cell proliferation and vasculogenesis to continue without the inhibitory effect of pericytes (51).

#### Angiopoietins

Angiopoietins are a group of glycoproteins which act as ligands for the tyrosine kinase receptors TIE (Tyrosine kinase with immunoglobulin-like and EGF-like domains). The TIE-2 receptor is exclusively expressed on the surface of endothelial cells (52). Angiopoietin-1 (ANG-1) is a ligand for the TIE receptors which is expressed by perivascular cells in response to PDGF-B stimulation (53). Activation of TIE-2 receptors causes stabilization of the vessel through the inhibition of apoptosis in endothelial cells, enhancing inter-endothelial connections and reducing endothelial layer permeability (54–56). ANG-2 is released mainly by endothelial cells and acts as an antagonist at the TIE-2 receptors preventing the anti-angiogenic activity of ANG-1 and thereby promoting angiogenesis (57).

#### Sphingosine-1-phosphate

Sphingosine-1-phosphate (S1P) is a sphingolipid metabolite released by perivascular cells (58) which acts on the endothelial differentiation gene-1 (EDG-1) receptor on the endothelial cell surface. S1P specifically promotes membrane expression and trafficking of the cell-cell adhesion protein N-cadherins to the cell membrane. N-cadherins localize to the peg and socket contacts between the endothelial and pericyte where it enhances these contacts (54, 59). Similar to PDGFR-β knockouts, loss of S1P results in absence of pericyte association with the vessels and subsequent vessel dilation and hemorrhage (54). Binding of the S1P also upregulates the expression of the inter-endothelial cell adhesion molecule VE-cadherin and down-regulates the expression of the pro-angiogenic ANG-2 thereby strengthening tight junctions between cells and increasing the stability of the developing vessel (36, 58–61).

#### Notch receptors and ligands

Notch receptors are transmembrane proteins which binds to Notch ligands which are also typically membrane bound structures, therefore, having an important role in mediating signaling between adjacent cells in direct contact. Binding of a Notch ligand causes the intracellular effector domain of the Notch receptor to be cleaved which travels to the nucleus where it acts as a transcription factor (62). Endothelial cells express the Notch ligands delta-like-ligand 4 (DLL-4), Jagged-1 (JAG-1), and Jagged-2 (JAG-2) and Notch receptors 1–4 while perivascular cells express Jagged-1 and Notch receptors 1–3 (63).

Notch receptors on the endothelial cells binds JAG-1 on the perivascular cells mediating contact inhibition by downregulating expression of VEGFR2 and upregulating VEGFR1 therefore desensitizing the cell to VEGF activation, (64, 65) reducing endothelial proliferation (66–68) and encouraging the formation of a mature endothelial cell phenotype (69). The expression integrin αvβ3 is increased and which causes adhesion of endothelial cells to von Willebrand factor in the basement membrane and strengthens the structure of the developing vessel (69).

Activation of Notch receptors in the perivascular cells upregulate the expression of PDGFR-β which promotes maturation of perivascular cells with increased expression of α-SMA (68, 70). It also enhances recruitment and attachment of perivascular cells to the endothelial layer forming more stable and mature vessels (71). Notch knockout mice have early embryonic lethality due to widespread vascular abnormalities (72).

### Extracellular matrix for vessel formation

In embryogenesis, the ECM and particularly the vascular basement membrane plays a vital role in controlling the development of the primitive vasculature by recruiting cells to the surrounding area and modifying their transcription profile depending upon the composition of the surrounding ECM (73). The vascular basement membrane is a structure of highly crosslinked insoluble materials including collagens, laminins, and fibronectin in which endothelial cells are embedded (74). This is formed by synthesis and deposition of these substances from both endothelial cells and perivascular cells which is upregulated following contact between these two cell types (2).

The basement membrane is not a static structure; it is continually being remodeled depending upon the conditions of the vessel and surrounding tissue. The changes in composition affect the endothelial cell behavior through interaction with integrins on the cell surface. MMPs and tissue inhibitors of metalloproteinases (TIMPs) catalyze this remodeling and are in constantly shifting equilibrium (75). Close interaction of endothelial cells with perivascular cells cause TIMP expression to predominate leading to inhibition of MMP and a more stable, type IV collagen rich and highly cross-linked basement membrane (76). In this state growth factors and signaling molecules including VEGF and TGF-β remain embedded in the basement membrane and are not available for the cell to access causing it to remain in a state of quiescence. In contrast, when the basement membrane is being assembled or disassembled by MMPs these growth factors are released and activate endothelial cells by exposing them to provisional matrix components, such as vitronectin, fibronectin, type I collagen, and thrombin. Additionally, growth factors including TGF-β and VEGF which are released from their latent bound states within the ECM and encourages endothelial proliferation and migration leading to sprouting and growth of the vessel (75, 77).

#### Cadherins

The cadherins, N-cadherin and VE-cadherin mediate the interaction between endothelial cells and surrounding cells and structures by linking the extracellular environment to the cytoskeletal framework of the cell (78). VE-cadherin is concentrated at adherens junctions, its extracellular component mediates cell-cell interaction with cadherins on other endothelial cells (79). The inhibition of this interaction with monoclonal antibodies results in increased permeability of the endothelial layer and apoptosis of endothelial cells (80). Congenital absence of VE-cadherin causes early embryonic lethality due to a lack of organized vasculature (81).

The intracellular components of the VE-cadherin complex; β-catenin, p120-catenin and plakoglobin act on various pathways to control gene expression, proliferation, and the cytoskeleton. Binding of VE-cadherin causes VEGFR2 to be internalized and degraded therefore mediating the contact inhibition seen in confluent endothelial cell culture (82). Interaction with catenin molecules causes alteration in the cytoskeleton on binding of VE-cadherin molecules which causes the endothelial cells to change shape in response to contact with neighboring cells. The expression of VE-cadherin is under the control of multiple growth factors including VEGF causing the phosphorylation of VE-cadherin and destabilization of the adherens junction (83, 84). This results in increased permeability of the endothelial cell layer and increased mobility of endothelial cells during angiogenesis.

N-cadherin molecules are positioned on the abluminal side of the endothelial cells mediating its contact with pericytes and disruption of this interaction with N-cadherin blocking antibodies impairs the pericyte-endothelial interaction. Although pericytes are recruited to the perivascular niche, they are weakly associated with the endothelial layer. Large extravascular cavities form and there is rupture of the endothelial layer, loss of polarity and extensive hemorrhage (85, 86).

## Dysfunctional multicellular interactions in disease

### Congenital disease

The failure of cell-cell interactions between endothelial cells and perivascular cells have been implicated in a variety of congenital and acquired diseases (Table 1). Approximately 80 per cent of rare diseases have a genetic basis, so identifying the specific mutations in underlying cell-autonomous pathways may provide insight into more common developmental and disease processes.

**Table 1:** Congenital syndromes of dysfunctional multicellularity.

**Disease**	**Genetic background**	**Cell types involved**	**Cardiovascular phenotype**	**References**
CADASIL	Notch3	Smooth muscle cells	Central nervous system arteriovenous malformations	(68)
Hajdu-Cheney syndrome	Notch2	Endothelial cells	Ductus arteriosus, atrial and ventricular septal defects Valve abnormalities	(87)
Adams-Oliver syndrome	Notch1 DLL4	Pericytes, Smooth muscle cells Endothelial cells	Hypoplastic aortic arch, middle cerebral artery and pulmonary arteries.	(88)
Singleton-Merten syndrome	Helicase C Domain 1 Dexd/H-Box Helicase 58	Endothelial cells Smooth muscle cells	Aorta calcification, subaortic stenosis	(89)
Hereditary haemorrhagic telangiectasia	Endoglin ALK1 SMAD3	Smooth muscle cells Pericytes Endothelial cells	Arteriovenous malformations and telangiectasia	(90)
Alagille syndrome 2	Notch2 JAG1	Smooth muscle cells Pericytes Endothelial cells	Atrial septal defect Pulmonary stenosis Tetralogy of Fallot Hypertension	(91)
Von Hippel-Lindau syndrome	Hypoxia-inducible factor-2 alpha, *VHL* tumor suppressor gene	Endothelial cells Smooth muscle cells Pericytes	Stage-specific changes in vessel branching and an advanced progression toward an arterial phenotype	(92)
Idiopathic basal ganglia calcification	PDGF-B PDGFR-β Type III sodium dependent phosphate transporter 2	Pericyte Endothelial cells	Perivascular calcium deposits Cerebral aneurysm Arteriovenous malformations	(93)

#### Hereditary haemorrhagic telangiectasia

Hereditary haemorrhagic telangiectasia (HHT) is an autosomal dominantly inherited condition caused by mutations in the ALK-1 receptors or Endoglin receptor (ENG) which is a co-receptor in the TGF-β signaling pathway (94, 95). It is characterized by large arteriovenous malformations (AVMs) in major organs including the lungs, liver, brain and mucosa. The large fragile vessels formed in AVMs are prone to bleeding resulting in presentations with epistaxis or intracranial hemorrhage and causing shunting between the arterial and venous circulation which can present as paradoxical emboli or high output heart failure (95). The loss of TGF-β signaling in endothelial cells reduced expression of N-cadherin which impaired the formation of heterotopic cell contacts with perivascular cells. This lack of cell-cell contacts results in further reduction in TGF-β activation from the surrounding ECM and therefore reduced TGF-β signals for the perivascular cell to encourage differentiation and attachment to the endothelial layer. This impaired recruitment of perivascular cells to the developing vessel and overactive proliferation of endothelial cells results in endothelial hyperplasia. These large dilated vessels with little perivascular cell coating develop into the arteriovenous malformations which characterize the disease (95–97). Similar phenotypes are seen in spontaneous cerebral AVMs with histological analysis demonstrating dilated vessels with segmental loss of smooth muscle cells and the internal elastic lamina. This phenotype is reproducible in mice by knockout of the ALK-1 and TGF-β receptor (98).

Currently emerging treatment for HHT is targeted at increasing the number of pericytes associated with the endothelial layer. Thalidomide is one such treatment which reduces the frequency and severity of epistaxis in patients with HHT (99). In mice, thalidomide has been noted to increase the number of pericytes surrounding vessels and improves their attachment to the endothelial cell layer thereby stabilizing the vessel which is likely to play a role in the reduced bleeding from AVMs. Thalidomide treated mouse retinas have increased expression of PDGF-B by endothelial cells (94) providing a potent chemotactic and maturation signal to neighboring pericytes.

#### Idiopathic basal ganglia calcification

Idiopathic basal ganglia calcification is a rare disorder, most commonly inherited in an autosomal dominant fashion. It presents with neurological and psychiatric abnormalities at a young age with associated areas of calcification within the basal ganglia of the brain. A loss of function mutations in PDGF-B is associated with this condition in humans (100). This is thought to cause reduced pericyte recruitment to cerebral vasculature which increases the permeability of the blood-brain barrier. Mouse models of the disease involving knockdown of PDGF-B function result in similar reduction in perivascular coverage of the cerebral vasculature and progressive brain calcification (100).

#### Notch mutations

Mutations in Notch receptor genes or ligands lead to a variety of congenital disorders in humans. Adams-Oliver syndrome is a rare inherited disorder, with varying degrees of defective vasculature defects including hypoplastic aortic arch, middle cerebral artery, and pulmonary arteries. The spectrum of vascular defects has been thought to be due to a disorder of vasculogenesis, perivascular dysfunction and abnormal vascular coverage during development (88, 101, 102). To support this idea, transient inhibition of Notch signaling in perivascular cells inhibited their differentiation and led to localized hemorrhages in newly forming vasculature during embryonic development (103). Alagille syndrome is a multi-systems inherited disease caused by loss of function mutations in JAG1 or NOTCH2 (91). Pulmonary artery involvement including hypoplastic pulmonary arteries and pulmonary stenosis are the common vascular manifestation of the disease. There is also a high rate of intracranial hemorrhage up to 16% suggesting the presence of fragile intracranial vessels (91).

CADASIL (cerebral autosomal dominant arteriopathy with subcortical infarcts and leukoencephalopathy) is a degenerative disorder characterized by early-onset strokes and dementia; it is caused by loss of Jag1 in the endothelium or defective Notch3 in vascular smooth muscle cells. This results in alterations of vascular smooth muscle cells; NOTCH1 and NOTCH3 mutant mice develop arteriovenous malformations and show hallmarks of CADASIL (68).

#### Tie mutations

Hereditary cutaneomucosal venous malformation is a rare condition which is inherited in an autosomal dominant fashion due to abnormalities in the TIE-2 receptor gene (*TEK*). This causes small and usually asymptomatic venous malformations mostly in the mucous membranes and face and much less common involving the internal organs (104). The mutation is a hyperphosphorylating mutation within the TIE-2 receptor which results in a patchy coating of perivascular cells along the vessel wall associated with hyperplasia of the endothelial layer producing large and dilated vessels (105–107).

### Acquire disease

#### Retinopathy

Diabetic retinopathy is a common condition affecting over one third of patients with diabetes worldwide (108). Chronic hyperglycaemia causes endothelial cells to release reactive oxygen species and inflammatory cytokines and reduces the expression of growth factors including PDGF-B (109). These changes coupled with direct effects of hyperglycaemia on the basement membrane and pericytes cause the vessel to become denuded of pericytes (110–112). This is one of the earliest histological findings in diabetic retinopathy which is followed by microaneurysm formation and oedema which can be detected clinically as the fragile neovasculature ruptures or leaks (113).

As described previously physical contact between pericytes and endothelial cells is required for activation of TGF-β and deficiency of TGF-β signaling impairs vessel maturity and increases permeability (114, 115). Additionally, ANG-1 released by pericytes has an important role in maintaining quiescence of the endothelial layer and reducing the permeability of the vessel. Blockage of the ANG-1 pathway using the competitive antagonist ANG-2 in normoglycaemic mice induces pericyte loss similar to diabetic retinopathy and subsequent endothelial hyperplasia. Models of diabetic retinopathy can also be induced in mice by reducing PDGF-B and TGF-β signaling in the absence of hyperglycaemia (114–117).

Pathological neovascularization also occurs in the setting of age-related macular degeneration (AMD). AMD is a condition where there is neovascularization of the macula of the retina which causes progressive blindness. Wet AMD is associated with macular oedema as the fragile and permeable vessels allow the leakage of fluid into the subretinal space (118).

The mainstay of treatment to date has been VEGF inhibition with monoclonal antibodies against VEGF which reduces the permeability of these vessel and induces regression of the vasculature. This treatment has significantly reduced the rates of progression and blindness associated with this condition (119). However, a proportion of patients become resistant to anti-VEGF treatment after an initial period of success. Pericytes have been implicated in providing survival signal to the developing blood vessels overcoming the inhibition of VEGF leading to ongoing angiogenesis within the macula. Combination of monoclonal antibodies against VEGF and PDGF have shown promise in pre-clinical trials to reduce the extent of neovascularization (120) and currently a trial of combination therapy is ongoing (121, 122). Similarly, treatments targeting other combinations of signaling molecules involved in this pathway are currently being trialed in diabetic retinopathy. This includes antibodies targeting both VEGF and Angiopoietin-2 (Clinicaltrials.gov-BOULEVARD, Clinicaltrials.gov-RUBY) and treatments targeted at VEGF, PDGF-B and FGF (clinicaltrials.gov-squalamine).

#### Cancer

One of the hallmarks of cancer is the ability to induce angiogenesis to augment the blood supply to the rapidly dividing cancerous cells (123). Therefore, targeting this pathway with inhibitors of angiogenesis has become an important target in oncological therapy. Monoclonal antibodies blocking VEGF and inhibitors of tyrosine kinase receptors have become a mainstay in cancer regimes for multiple indications (124). However, after a period of treatment, a proportion of cancers become resistant to the anti-VEGF treatment and escape this inhibition. Thus, further efforts have been invested in developing therapies to target the pericyte-endothelial cell interaction to prevent this resistance developing. Less selective tyrosine kinase inhibitors with action against multiple tyrosine kinases has shown promise in this area. Sunitinib targets the VEGF and PDGF receptors and is a potent inhibitor of angiogenesis. It reduces the coverage of pericytes along the developing vessel causing reduction in angiogenesis overall and therefore improved survival in renal cell cancers (125, 126). Preventing migration of perivascular cells to the perivascular niche of developing cancer vasculature reduces the survival signal to endothelial cells leading to apoptosis and preventing further angiogenesis (127).

Manipulation of TIE2/Angiopoietin pathway has also been targeted in preclinical models of renal cell cancer by using antibodies which selectively block the effect ANG-2, therefore, potentiating the effects of ANG-1 which will stabilize the vasculature and prevent further angiogenesis and therefore limit further growth of the tumor (128). This is currently being trialed in phase 1 human trials (129).

## Multicellular interactions in culture

Modeling rare vascular genetic disease *in vitro* is possible using pluripotent stem cell technology which provides insights into the mechanisms involved in these diseases. The use of multicellular constructs provides a more analogous environment to the *in vivo* environment than can be replicated by the exogenous application of growth factors as it is able to model and regulate a greater variety of interaction between cells in a more physiological manner.

### Endothelial cells

The earliest stage of *in vitro* blood vessel formation involves expansion of endothelial cells in culture to form a network of tubular structures replicating the early steps of vasculogenesis in the embryo. Multiple cell types and sources have been used to provide endothelial cells for this purpose. Fully differentiated primary endothelial cells have been used, most commonly these are sourced from the human umbilical vein (HUVEC). These cells already have a fully differentiated endothelial phenotype and therefore will immediately begin forming tubular structures in culture (35). However, use of these cells is limited by replicative senescence which develops after limited passages in culture making them sub-optimal for large-scale replication. Less well differentiated cells including late outgrowth endothelial cells (OEC) or multipotent cells such as mesenchymal stem cells (130) show greater replicative potential and are also partially differentiated toward vascular lineage, therefore, require less manipulation of growth factors to produce fully differentiated endothelial cells.

Pluripotent cells including embryonic stem cells or inducible pluripotent stem cells have attracted most interest recently (131, 132). The ability to produce more than one cell type from the a single cell source enables the development of complex vascular structures incorporating endothelial cells and supporting cells from the same cell source. This raises the possibility of individualized regenerative medicine approaches being possible in the future and additionally, makes it possible to create models of inherited genetic disease states *in vitro* by inducing pluripotency in cells of patients with specific genetic diseases and creating models of vasculogenesis using these cells.

There are three main methods used to differentiate pluripotent cells toward endothelial phenotype (131). Each of these methods attempt to replicate parts of the embryonic development of endothelial cells and vascular networks. The first method requires embryoid body formation which are three dimensional aggregates of pluripotent stem cells which form in suspended culture. These replicate the blastocyst formation seen *in vivo* (133, 134) and begin to differentiate with all three primitive germ layers represented; ectoderm, mesoderm and endoderm. Aggregates of cells expressing early endothelial markers developed within the embryoid body resembling blood islands (135) and these can be expanded and selected for by plating on suitable matrix with the addition of exogenous growth factors (136, 137).

The second commonly used method is co-culture of pluripotent stem cell with a feeder layer of mesenchymal cells such as bone marrow stromal cells. The feeder later provides growth factors to the stem cells encouraging differentiation toward mesoderm and expression of key endothelial markers (8, 138, 139). This also recapitulates the embryonic development of endothelial cells by providing Indian hedgehog (IHH) signaling which acts through BMP4 to upregulate endothelial markers such as VE-cadherin, CD31 and VEGFR2. These effects are abolished by neutralizing either IHH or BMP4 signaling and IHH signaling is rescued by exogenous BMP4 (8).

The third method is by 2D culture of inducible pluripotent stem cells on culture plates coated with protein substrate and culture media enriched sequentially with specific growth factors to direct differentiation toward mesodermal lineage and then endothelial lineage. The two most common protocols used to differentiate pluripotent cells toward endothelial lineage have numerous similarities and both attempt to recreate the embryonic development in the blastocyst. Mesodermal differentiation in the embryo relies on BMP-4, nodal and Wnt signaling pathways (132). *In vitro* supplementation of the media with BMP-4 and inhibitors of GSK3β, which upregulates the Wnt/B-catenin pathway, or Activin A which acts on nodal receptors are used to achieve this mesodermal transition (29, 140–142).

Following mesodermal induction, the addition of VEGF to the media is needed to start endothelial differentiation. Activators of the protein kinase A pathway such as forskolin or 8-bromo-cAMP appears to enhance the effects of VEGF and produce a greater purity of endothelial cells (29, 143, 144). The endothelial cells formed from these differentiation procedures have multiple markers of endothelial lineage including VE-cadherin, CD31 and von Willebrand factor, however, there remain a proportion of cells which differentiate toward other lineages and therefore to obtain a pure culture of endothelial cells requires cell sorting and re-plating of endothelial cells.

### Perivascular cells

The source of perivascular cells used in multicellular culture also varies, however, these are typically mesenchymal in origin. This includes aortic smooth muscle cells, adipose derived stem cells, osteoblasts (145) and mesenchymal stem cells (146). Pericytes have also been developed using embryoid body method from pluripotent stem cells. Similarly, to the endothelial cell protocols following formation of the embryoid bodies in suspended culture they can be dissociated and sorted to using cell surface markers to identify a population of pericytes which can then be plated and expanded (147).

It is also possible to produce perivascular cells from iPSC by manipulation of growth factors in the growth media. As with endothelial cells, the first step requires induction of mesoderm which is followed by supplementation with PDGF-B along with TGF- β or Activin A to direct differentiation toward a perivascular cell fate. This is highly efficient at creating a culture of perivascular cells which can be used in co-culture experiments with endothelial cells. (140, 148) Orlova et al used a single source of hiPSC and exposed them to a short period of supplementation with VEGF to begin vascular specification which yielded a mixed population of CD31+ and CD31- cells. Following cell sorting the endothelial and perivascular cells were able to be produced from the CD31+ and CD31- fractions, respectively (149). In co-culture, the perivascular cells were able to control sprouting and proliferation of the endothelial cells in a TGF-β dependent manner similar to that seen in the developing embryo.

### Co-culture

Combining endothelial cells and pericytes to create multicellular models of vasculogenesis can recreate a greater degree of the complexity of intracellular signaling seen *in vivo* than is provided by supplementing the media with supra-physiological levels of growth factors. Intrinsic intercellular regulatory mechanisms which are as yet incompletely understood and therefore difficult to replicate may provide a more physiological environment in which to model vasculogenesis.

Culture of mesenchymal stem cells (MSC) alongside the developing vascular network of endothelial cells increases the survival of vessels and helps to maintain these vessels in long-term culture (150). This coculture has been shown to upregulate VE-cadherin on the cell surface of endothelial cells and reduce the rate of proliferation and apoptosis of endothelial cells (151, 152). This results more stable, less permeable cells which form a more durable vascular network. Chen et al used co-culture of HUVEC and mature perivascular cells obtained from skeletal muscle to create vascular networks in Matrigel plugs (28). The inclusion of perivascular cells supported the formation of complex capillary structures within the plugs which was not seen with culture of endothelial cells alone (28).

Additionally, Koike and colleagues produced a model of vasculogenesis by combining HUVEC with mesenchymal stem cells in a 3D type 1 collagen and fibronectin construct and implanting these into mice. The implantation of HUVEC alone formed tubules initially but it was unable to support perfusion and subsequently, these tubules regressed. In contrast during co-culture experiments, the mesenchymal precursor cells migrated to a perivascular position started to express perivascular cell markers including α-SMA and produced stable long-lasting blood vessels which were able to support flow for over 60 days (150).

Melero-Martin and colleagues have produced similarly robust vascular networks in Matrigel plugs by using blood and cord blood derived OEC and MSC derived from the bone marrow. OEC and MSC have a greater replicative capacity than primary cell types and can be harvested from the same donor from blood and bone marrow. This enables sufficient quantities of genetically identical cells to be produced which enables more accurate modeling of inherited diseases and has potentially be used in regenerative medicine to vascularize tissue engineered grafts (153).

The development of inducible pluripotent stem cells (iPSC) by Takahashi and colleagues (154) provided further tools to build multicellular constructs which could now be derived from a single cell without many of the ethical difficulties which surrounded embryonic stem cells. Samuel et al developed endothelial and mesenchymal precursor cells in parallel from human iPSCs and by combining these cells in culture were able to form robust long-lasting functional blood vessels when implanted in mice (155). This parallel development of endothelial cells and the supporting perivascular cells was a step forward, however, still requires cells to be dissociated and sorted using flow cytometry before being plated and expanded.

Kusuma et al have started to overcome this difficulty by creating a bipotent population of cells from hiPSC which are able to differentiate toward endothelial and perivascular cell lineage (156). The difficulty with this approach *in vitro* is that proliferation is inhibited when perivascular cells and endothelial cells come into contact. Therefore, they initially focused on developing VE-cadherin positive endothelial cells. They used high concentrations of VEGF and inhibited TGF-β signaling which causes perivascular cell differentiation and mediates contact inhibition of endothelial cells (15, 157, 158). This created two populations of early vascular cells both of which expressed CD146^+^ and CD105^+^ and were negative for the haematopoietic marker CD45. However, the endothelial precursors expressed VE -cadherin while the perivascular precursors expressed PDGFRβ. When plated in hydrogel these cells were able to self-assemble into tubular-like structures of endothelial cells with perivascular cells surrounding these vessel forming a multicellular vascular network (156). Although these networks were not durable and typically regressed within approximately 2 weeks it provided a significant step forward in developing engineered multicellular vascular networks (159).

Use of patient-specific hiPSC-derived endothelial cells is now being used for disease modeling some of the inherited vascular disorders. The model of CADASIL is one of the first attempts to gain a better understanding of the developmental biology and cross-talk of endothelial cells and perivascular cells with *NOTCH3* mutations. Similarly, NOTCH1 mutations have also been studied with pluripotent stem cell platforms (160). This *in vitro* approach may also facilitate the development of therapeutic gene editing interventions for vascular malformations and arteriopathies in the future.

The use of three cells together in culture has recently introduced and this extra complexity appears to have further benefits to promote stable vasculature. Caspi and colleagues used coculture of HUVEC, embryonic fibroblasts and embryonic stem cell derived cardiomyocytes on a biodegradable scaffold. The embryonic fibroblasts differentiate into perivascular cells and produced increased complexity of vascular networks. Additionally, there was upregulation of several growth factors in this model including VEGF, PDGF-B and ANG-1 compared to single cell culture and the endothelial cell survival was higher (161). Similarly, bone grafts have been produced by combining HUVEC and CD146+ perivascular cells with human inducible pluripotent mesenchymal stem cells in a calcium phosphate cement. This triculture demonstrated more complex vascular networks compared to culture with mesenchymal stem cell and endothelial cells alone. There was more rapid induction of VEGF signaling and there was improved bone mineral density all of which suggested benefits from combining multiple cell types (162).

## Conclusions

Development of functional blood vessels requires precise interaction between the tubule forming endothelial cells and the surrounding environment including growth factors, ECM and perivascular cells. The crosstalk between perivascular and endothelial cells is complex and incompletely understood. This cross talk plays a vital role in normal blood vessel development and homeostasis while abnormalities in this relationship have been implicated in multiple congenital and acquired diseases.

Knowledge of the molecular process involved in vasculogenesis and angiogenesis provides insight into the pathophysiological mechanism multiple vascular diseases including diabetic retinopathy and cancer. Therapies targeting the interaction between endothelial cells and neighboring perivascular cells are already in use or in late stages of development (94, 121). However, an in-depth understanding of the crosstalk between these cells will allow the development of more accurate vascular models to enable the identification of novel drug targets and further guide drug development.

Improved understanding of vascular development will also provide useful in the field of regenerative medicine where ensuring perfusion of stem cell derived tissue is one of the key challenges in producing viable implantable grafts. The development of vascular networks using multiple cell types is being used increasingly commonly and appears to provide greater complexity, stability and durability of the vasculature. Approaches using pluripotent cells are particularly intriguing given the possibility to develop these structures from a single cell source (156). This has the potential to develop more accurate models of vascular disease as well as personalized regenerative medicine applications.

## Author contributions

All authors listed have made a substantial, direct and intellectual contribution to the work and approved it for publication.

### Conflict of interest statement

The authors declare that the research was conducted in the absence of any commercial or financial relationships that could be construed as a potential conflict of interest.
